# Successful outcome of a pregnancy in a woman with advanced cirrhosis due to hepatitis B surface antigenemia, delta super-infection and hepatitis C co-infection: a case report

**DOI:** 10.1186/1752-1947-1-96

**Published:** 2007-09-20

**Authors:** Amna Subhan, Shahab Abid, Wasim Jafri

**Affiliations:** 1 Department of Medicine, Aga Khan University Hospital, Karachi, Pakistan

## Abstract

Pregnancy in women with advanced liver disease is rare. In this paper we described the case of a successful pregnancy in a young woman with advanced cirrhosis due to hepatitis B surface antigenemia, hepatitis delta super-infection and Hepatitis C co-infection. A brief review of the medical literature on pregnancy in women with cirrhosis is also presented.

## Introduction

Pregnancy is uncommon in women with advanced cirrhosis and is associated with an increased risk of complications such as bleeding from esophageal varices, liver failure, and hepatorenal syndrome [1-4]. Maternal deaths have been reported in advanced cirrhosis mainly due to variceal bleeding [4]. Spontaneous abortion and increased risk of premature childbirth or stillbirth have been reported in 15–20% of pregnancies in women with cirrhosis [5]. We are reporting the case of a successful pregnancy outcome of a woman with decompensated cirrhosis, affected by hepatitis B, C and D viruses. To the best of our knowledge this is the first report of pregnancy in a mother who had combined hepatitis B antigenemia, hepatitis delta and hepatitis C infection.

## Case report

**Figure 1:**
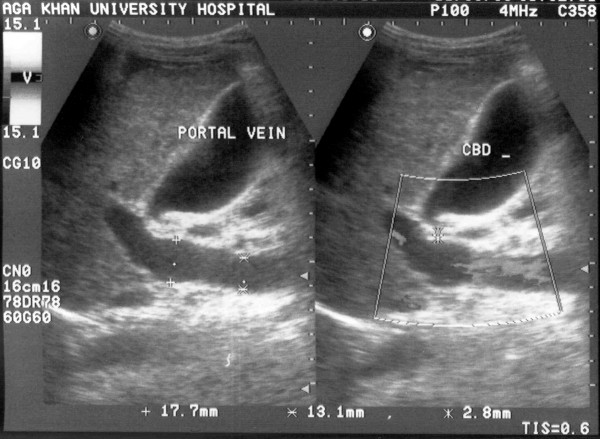
Ultrasound upper abdomen showing portal vein of 17.7 mm, coarse liver parenchyma.

**Figure 2:**
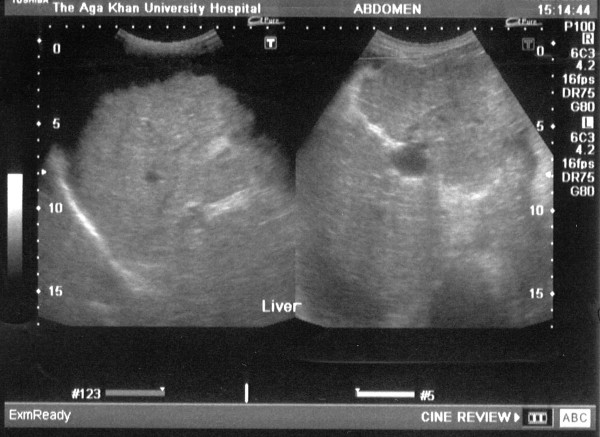
Ultrasound upper abdomen showing coarse liver parenchyma, irregular margins of liver, parahepatic ascities.

**Figure 3:**
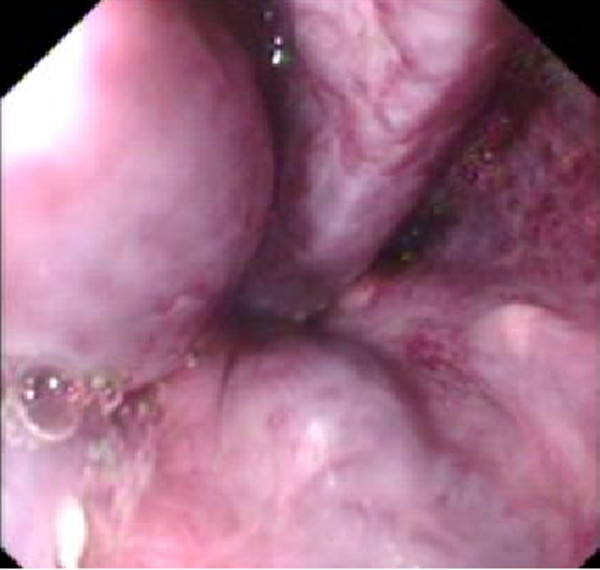
Endoscopic view of large esophageal varices with cherry red spots.

A 32 year old mother of two children presented at 16 weeks gestation with abdominal distention and edematous legs. There was no history of hematemesis, melena or altered mental status. Physical examination revealed pallor, spider telangectesia on the arms, palmer erythema and pedal edema. Her abdominal examination showed splenomegaly (to the level of the umbilicus) and moderate ascities. There was no clinical evidence of portosystemic encephalopathy. Her pulmonary, cardiovascular and neurological examinations were unremarkable. Investigations revealed hemoglobin of 8.5 gm/dl with peripheral smear suggestive of microcytic hypochromic anemia, total leukocyte count of 4200/mm^3 ^(63% polymorphs and 32% lymphocytes), platelets 40,000/mm^3^, total billirubin 2.0 mg/dl, serum glutamic oxaloacetic transaminase 74 (8–32) IU/L, serum glutamic pyruvic transaminase 41 (Normal 3–33) IU/L, GGT of 61 IU/L and alkaline phosphatase 81 (29–132) IU/L, serum albumin 2.3 gm/dl and prothrombin time 15.6 seconds (control 12 seconds). She had normal renal function and electrolytes. Abdominal and pelvic ultrasound revealed a shrunken liver, massive spleenomegaly, dilated portal vein, moderate ascities (fig 1 &2) and a 16 week viable fetus. Ascitic fluid analysis showed SAAG (serum ascitic albumin gradient) of >1.1 without any evidence of spontaneous bacterial peritonitis. She had positive HBsAg, HbeAb, HDV IgG, Anti-HCV antibody and HCV RNA in her serum. However, HbeAg and HBV DNA were not detected in her serum. Upper gastrointestinal endoscopy showed grade III esophageal varices (Fig 3) and severe portal gastropathy but no gastric varices. Prophylactic banding of esophageal varices was performed on two occasions (at 17^th ^and 23rd week of gestation). She was given propranolol 10 mg trice a day and spiranolactone 200 mg daily. She underwent therapeutic paracentesis of massive ascities at 28^th ^week of gestation. Despite elaborate preparation for a planned vaginal delivery under controlled circumstances, the patient underwent an unanticipated rapid labor and delivered a baby boy at a local facility near her home during the 36th gestational week.

The patient and the baby did not have any complications in the postpartum period. Her ascites was controlled with diuretics. Surveillance endoscopy performed one year following delivery showed small esophageal varices and mild portal gastropathy. The baby was given active and passive immunization against hepatitis B. At the age of 18 months the baby's blood was tested for hepatitis B and C. Serology showed undetectable Anti-HCV antibody and Anti-HDV Ig-G. However, HBsAg was found to be positive with normal ALT and undetectable HBV DNA.

## Discussion

Infertility is common even in mild forms of chronic liver diseases. Advanced cirrhosis increases the risk of maternal and fetal morbidity and mortality [1,2]. In such cases the stage of the liver disease is the most important determinant of the outcome of the pregnancy [1,2,6,7].

In contrast to cirrhosis due to autoimmune etiology or Alcoholic Liver Disease, the outcome of pregnancies in women with other types of chronic liver disease, especially of viral etiology, is poorly reported and therefore uncertain [8]. To date there has been no reported case series related to the outcome of the pregnancy in patients with decompensated cirrhosis due to viral etiology. Our patient had had exposure to three hepatotropic viruses i.e. HBV, HCV & HDV and had a successful outcome of pregnancy.

Maternal death rate in women with cirrhosis is reported to be 10.3% to 18% with massive gastrointestinal bleed as the commonest cause of death [1,3] and liver failure as the next most frequent cause of death [2]. In a review of 117 pregnancies a term pregnancy without maternal complications was achieved in 50% of cases while deterioration in liver function tests was observed in 44.4% of cases. Hematemesis occurred on 24 occasions and was responsible for maternal deaths in 4 % of patients [9].

Portal hypertension due to cirrhosis compounds the physiological increase in circulating blood volume, elevation in portal pressure and added pressure from the gravid uterus on the inferior vena cava and can result in massive bleeding. It is most common during the second trimester with 20–27% chance of bleeding from esophageal varices which is amplified to 62–78% if there are demonstrable varices [1-4,7]. Therefore, it is mandatory to assess such patients for portal hypertension, which can be done by indirect evidence, such as the presence of esophageal varices, abdominal collateral veins, hypersplenism and ascites. Endoscopic variceal band ligation or sclerotherapy, portosystemic shunting, esophageal transection and beta-blockers are the therapeutic options for such patients [2,3]. Screening EGD was done in our case and prophylactic variceal band ligations were applied on two occasions. Fortunately despite thrombocytopenia and abnormal coagulation she did not bleed during the pregnancy from her varices.

There is an increased rate of spontaneous abortion, premature birth and perinatal deaths in pregnant woman with advanced cirrhosis. Poor fetal prognosis is usually explained by poor condition of the mother in decompensated patients. Though, infants born alive generally remained well [2,3].

In a controlled setting vaginal delivery is usually safe and early forceps delivery or vacuum extraction should be considered to prevent any rise in portal pressure due to prolonged straining during labor [2,3]. Women with cirrhosis generally tolerate laparotomy poorly; therefore the option for caesarean section should be availed with care and caution. Our patient had an uncomplicated vaginal delivery without any massive bleeding. She did not have further hepatic decompensation, sepsis or any other complication. The baby boy had normal Apgar scores and birth weight and had normal growth up to the end of follow up at 18 months of age.

In short, the data related to the optimal management and outcome of pregnancy in women with decompensated cirrhosis secondary to viral etiology is limited. Whether to advise a pregnancy to a woman with decompensated cirrhosis is a difficult question to answer. However, careful overall assessment of the severity of the liver disease as well as of the patient's psychological status and desire for children should lead logically to a resolution of these issues on a case by case basis. With careful monitoring and advanced management, successful pregnancy with a good outcome is a good possibility. The excellent outcome of the pregnancy in our patient is encouraging and supports this opinion.

## Competing interests

The author(s) declare that they have no competing interests.

## Authors' contributions

AS performed the literature search and wrote the first draft of the manuscript. SA obtained the patient consent. SA and WJ proof read the case report and finalized it.

## Consent

Written consent was obtained from the patient for publication of this case report.
